# Computational modeling of the effects of auditory nerve dysmyelination

**DOI:** 10.3389/fnana.2014.00073

**Published:** 2014-08-01

**Authors:** Angus M. Brown, Martine Hamann

**Affiliations:** ^1^School of Biomedical Sciences, Queens Medical Centre, University of NottinghamNottingham, UK; ^2^Department of Cell Physiology and Pharmacology, University of LeicesterLeicester, UK

**Keywords:** myelin sheath, hearing loss, node of Ranvier, conduction velocity, conduction block, deafness, myelin domains, action potential

## Abstract

Our previous study showed that exposure to loud sound leading to hearing loss elongated the auditory nerve (AN) nodes of Ranvier and triggered notable morphological changes at paranodes and juxtaparanodes. Here we used computational modeling to examine how theoretical redistribution of voltage gated Na^+^, Kv3.1, and Kv1.1 channels along the AN may be responsible for the alterations of conduction property following acoustic over-exposure. Our modeling study infers that changes related to Na^+^ channel density (rather than the redistribution of voltage gated Na^+^, Kv3.1, and Kv1.1 channels) is the likely cause of the decreased conduction velocity and the conduction block observed after acoustic overexposure (AOE).

## Introduction

The rapid conduction of action potentials in both the central nervous system (CNS) and peripheral nervous system (PNS) depends on the myelin sheath around neuronal axons. Myelin plays a passive role by insulating axons. Myelin also plays an active role, allowing for saltatory conduction as action potentials regenerate via active, voltage dependent Na^+^ and K^+^ ion-specific conductances at the intermittent, non-myelinated nodes of Ranvier (Ranvier, 1871; Huxley and Stampfli, 1949; Rasband and Trimmer, 2001). The fundamental importance of myelin is highlighted in demyelinating diseases where the speed of conduction along axons is altered and sensory, motor and/or cognitive ability is severely compromised (Nave, 2010). The auditory nerve (AN) contains mainly myelinated axons projecting from type I spiral ganglion neurons to brainstem cochlear nuclei (Toesca, 1996). We recently showed that acoustic overexposure (AOE) leading to hearing loss also triggered notable morphological changes at AN myelin sub-domains such as nodes of Ranvier, paranodes and juxtaparanodes (Poliak and Peles, 2003) that were associated with the decreased conduction velocity (Tagoe et al., 2014). Specific changes involved a decreased number of lamella wraps and hence myelin thickness, a marked elongation of the nodes and juxtaparanodes, and a retraction of the paranodes (Tagoe et al., 2014). Precise localization of axonal ion channels is crucial for proper electrical and chemical functions of axons. It is unknown whether AOE-induced deficit in conduction velocity (Tagoe et al., 2014) is due to re-distribution of voltage-gated channels from specific myelin sub-domains. At the nodes of Ranvier, clusters of voltage-gated sodium channels facilitate saltatory conduction of action potentials (Rasband and Trimmer, 2001; Hedstrom and Rasband, 2006; Leterrier et al., 2010). Kv3.1 voltage-gated potassium (Kv) channels contributing to the ability of auditory neurons to fire at high frequencies (Kanemasa et al., 1995; Macica et al., 2003) are also clustered at the nodes (Devaux et al., 2003). Kv1.1 voltage-gated potassium (Kv) channels reduce action potential jitter (Gittelman and Tempel, 2006) and determine action potential threshold (Brew et al., 2003; Oertel et al., 2008), are clustered in the juxtaparanodal regions (Gu and Gu, 2011). Here we modeled changes in the AN conduction properties after AOE based on our recent data (Tagoe et al., 2014) and tested whether the decreased conduction velocity observed after AOE (Tagoe et al., 2014) could also be due to the redistribution of voltage gated Na^+^, Kv3.1 and Kv1.1 channels.

## Methods

### Computer simulations

**Figure 1:**
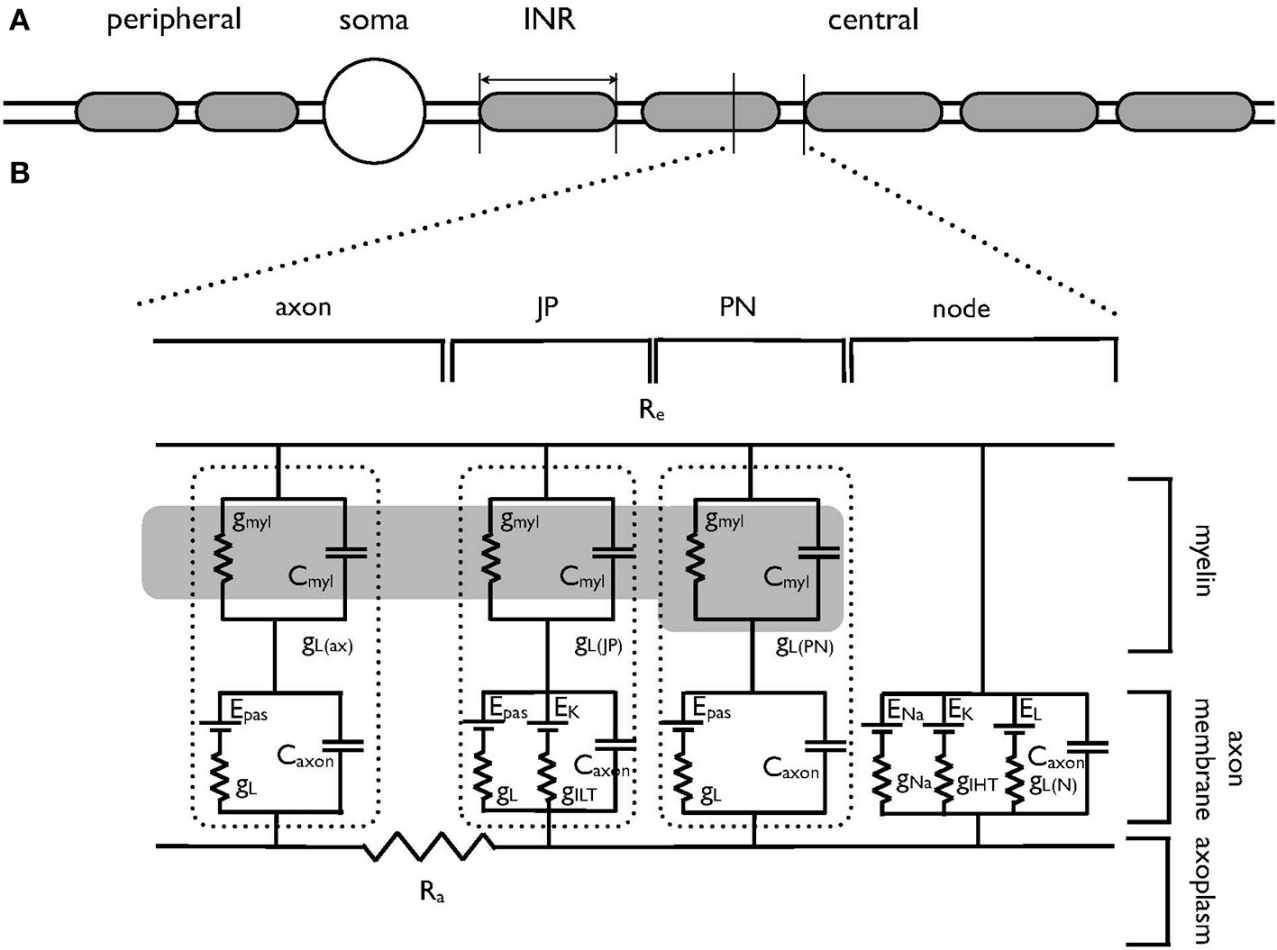
**Schematic model of the auditory nerve. (A)** The auditory nerve fiber is divided into three distinct regions. The peripheral axonal region terminates in the organ of Corti. The soma resides in the spiral ganglion. The central axon projects to the cochlear nucleus, is myelinated with internodal myelinated regions modeled as 100 μm in length. **(B)** Equivalent circuit of the central portion of the auditory nerve, which has been adapted from an existing model of corpus callosum axon (Tagoe et al., 2014). The morphological and electrical values are contained in Tables 1, 2 respectively. The axon is divided into the nodal region and the internodal region. The internodal region is subdivided into the paranodal (PN) juxtaparanodal (JP) and axonal regions (axon). The nodal region expresses the voltage-dependent conductances of the I_Na_ (g_Na_) and I_HT_ (g_IHT_) currents as well as a leak current (g_L_). Gi_LT_ is expressed in the JP. The axolemma of the internodal regions expresses a leak current (g_L_), as does the overlying myelin (g_myl_). The dotted lines enclose the leak and capacitative properties of each INR component. Internal resistance (R_a_) is constant throughout the model and external resistance (R_e_) is zero. The dark region represents the myelin, and only one PN and JP abutting the node are shown for clarity. g_myl_ and C_myl_ are the passive conductance and capacitance across the myelin, respectively. g_L_ is the passive conductance, g_L(N)_ refers to the passive conductance at the node, E_pas_ is the reversal potential for the passive conductance (V_L_), and E_Na_ and E_K_ are the reversal potentials for the Na^+^ current and ILT and IHT respectively.

Simulations were carried out using Neuron 7.1 (Hines and Carnevale, 1997) and were based on previously published morphological data on the AN (Tagoe et al., 2014). Briefly, the AN was modeled as a single axon unsheathed by myelin lamellae in a manner similar to that described previously (Kolaric et al., 2013). The axon comprised narrow nodal regions separated by larger internodal regions (INR, Figure 1A). Passive electrical properties were based on data from corpus callosum axons and oligodendrocytes (Bakiri et al., 2011) and voltage dependent conductances were based on previous studies including an existing model of the AN (Kanemasa et al., 1995; Macica et al., 2003) (Figures 1A,B). Current densities were derived from values in Kanemasa et al. (1995) and Macica et al. (2003) where maximum current amplitude was given relative to a cell capacitance (Cm), based on a specific Cm of 1 μF.cm^−2^. The simulated action potentials were computed using backward Euler integration with a time step of 10 ms. Our morphological measurements lack a value for the internodal length, thus we chose an intermediate value from two existing models of AN (Rattay et al., 2001; Smit et al., 2010) of 100 μm.

### Morphological values

**Figure 2:**
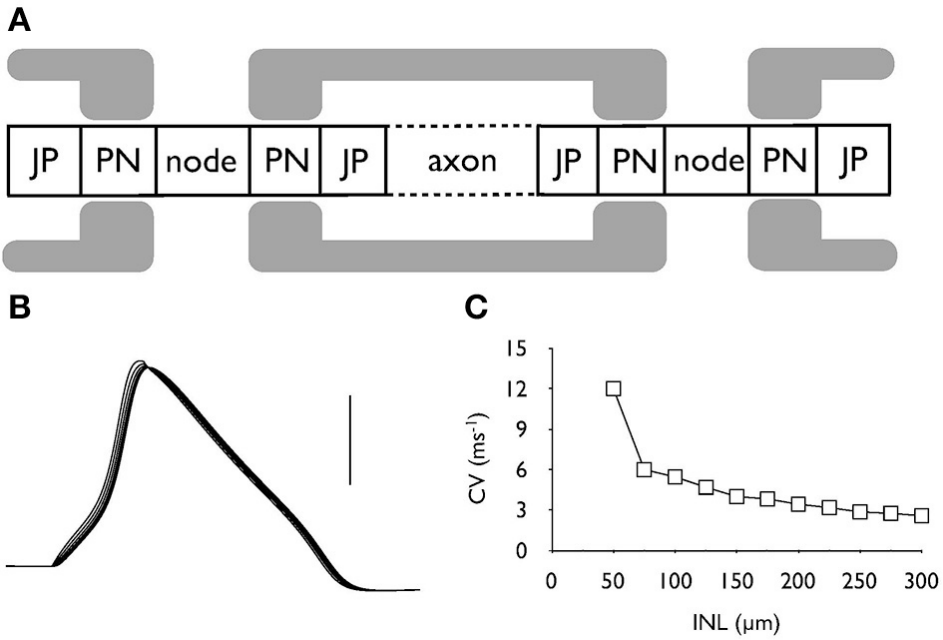
**Action potential conduction along the central portion of the auditory nerve. (A)** Schematic model of the axonal compartments in the control auditory nerve model. **(B)** Evoked action potentials recorded at six successive nodes illustrating action potential conduction along an axon. Scale bar is 50 mV and the duration of the recording is 2 ms. **(C)** The conduction velocity decreases as internodal length (INL) is increased from the control value of 100 μm.

The morphological dimensions of the axonal compartments have recently been published (Tagoe et al., 2014). The model of the control axon comprised alternating nodal and internodal regions (INR). The INRs were subdivided into paranodal (PN), juxtaparanodal (JP) and axonal compartments (Figure 2A), such that each INR comprised two PNs, each abutting consecutive nodal regions, two JP regions located between the PN and a central axonal portion (Figure 2A). The dimensions of the compartments are contained in Table 1. AOE changed the nodal length and diameter from 1.3 to 6.15 μm and 0.8 to 1.28 μm, respectively; the PN length and diameter from 2.34 to 1.52 μm and 0.75 to 1.23 μm respectively and the JP length and diameter from 5.14 to 6.23 μm and 2.13 to 1.32 μm respectively (Tagoe et al., 2014). The model contained a fast sodium current (I_Na_), a high threshold (I_HT_) Kv3.1 potassium current, a low threshold (I_LT_) Kv1.1 potassium current and a leak current (I_L_). We assumed that the current density for I_Na_ and I_HT_ in the nodal region were 6.6 mS.cm^−2^ and 1.98 mS.cm^−2^, respectively, and the value for I_LT_ in the PN was 2.13 mS.cm^−2^ (Kanemasa et al., 1995).

**Table 1:** **Morphological parameters of the auditory nerve in control and in the two simulated AOE conditions**.

	**Node**	**n1**	**PN**	**JP**	**j1**	**Axon**
**CONTROL**						
l (μm)	1.3	–	2.34	5.14	–	10.46
d (μm)	0.8	–	0.75	2.13	–	1.25
**AOE1**						
l (μm)	6.15	–	1.52	6.23	–	9.79
d (μm)	1.28	–	1.23	1.32	–	1.25
**AOE2**						
l (μm)	1.3	2.425	2.34	5.14	1.09	9.79
d (μm)	1.28	1.28	1.23	2.13	1.16	1.25

**Figure 3:**
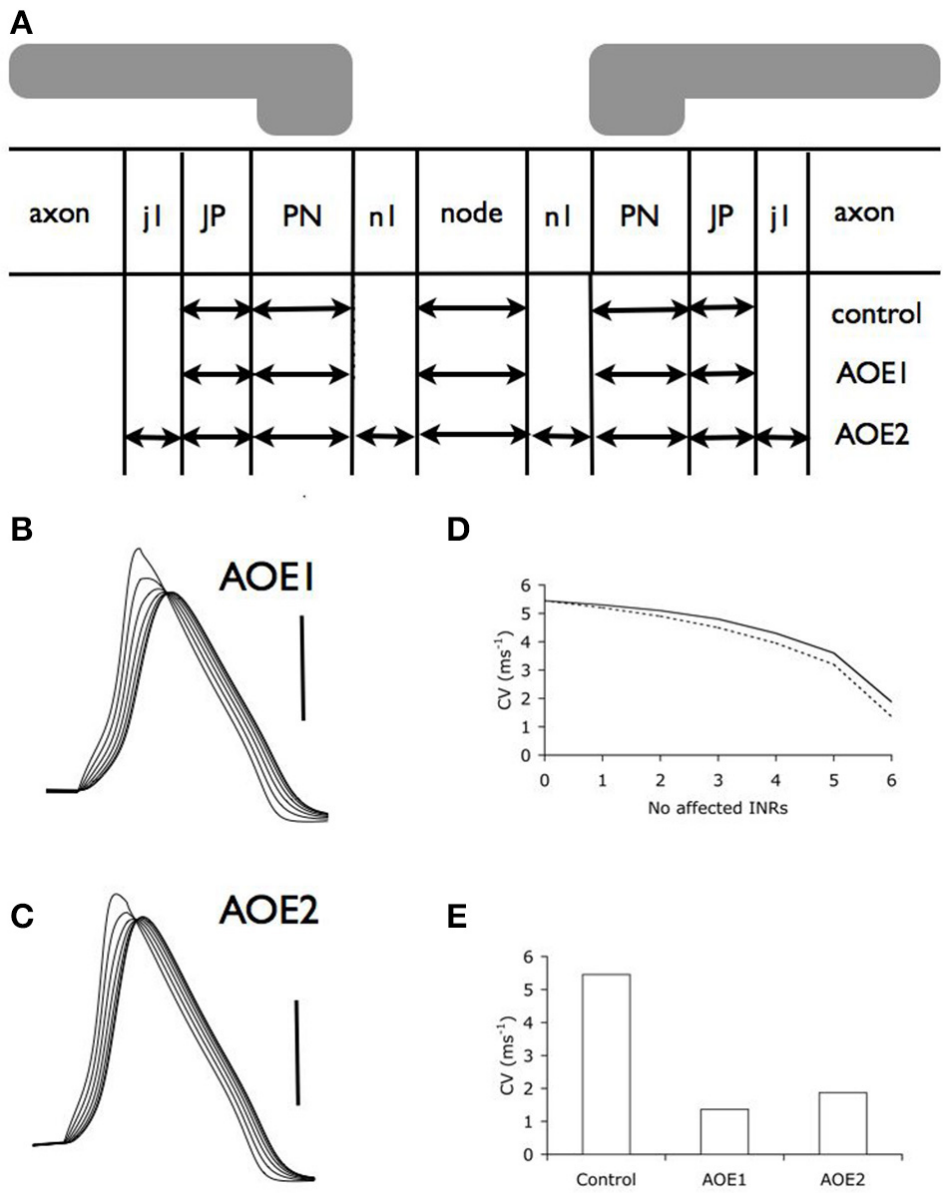
**Auditory overexposure decreases conduction velocity**. **(A)** Schematic model of the central portion of the auditory nerve after AOE, illustrating the morphological changes incurred by the nerve. See Table 2 for dimensions of compartments. **(B,C)**. Action potentials evoked from six successive nodes after AOE1 **(B)** or AOE2 **(C)** treatment demonstrated a decrease in the conduction velocity. Scale bars 50 mV in **B** and **C** and duration of recording is same as Figure 2B. **(D)** Incremental decrease in conduction velocity as more INRs are affected by AOE-induced dysmyelination in AOE1 (dotted line) and AOE2 (line) models. **(E)** Conduction velocity (with an INL of 100 μm) decreases as a result of both AOE1 and AOE2 (see Methods for details).

Morphological dimensions of the various compartments illustrated in Figures 2A, 3A, where l and d refer to length and diameter and p/j/a refer to paranodal, juxtaparanodal and axonal compartments. Axon dimensions are taken from previously published data Kolaric et al. (2013). The length indicates the compartments length of the axon used in the model with 20 compartments per internodal length.

**Table 2:** **Passive properties of the auditory nerve in control and after AOE**.

	**Control**	**AOE**
Cm _p/j/a_ (μF.cm^−2^)	0.0184	0.0290
Cm _node_ (pF)	0.0327	0.247
Cm _INR_ (pF)	0.136	0.167
gL _node_ (mS.cm^−2^)	0.2	0.2
gL _p/j/a_ (μS.cm^−2^)	10.89	20.3

*Passive properties of nodal and INR compartments in control and AOE treated nerves. p/j/a refers to paranodal, juxtaparanodal and axonal regions, all of which express the same values. G refers to conductance and Cm to capacitance. The capacitance and leak current were calculated for each individual INR and node demonstrating that AOE causes an increased INR capacitance due to decreased myelin wraps and increased nodal capacitance and leak current*.

We used these morphological changes to determine conduction properties under two conditions. Firstly, we modeled the effect of altering current distributions such that the compartments resulting from AOE treatment contained the same number of channels, but the density of these channels was altered to reflect an even distribution of the channels along the altered nodal, PN and JP length (AOE1 condition illustrated in Figure 3A). This condition named AOE1 resulted in morphological values contained in Table 1. Secondly, we modeled the axon as if the channels had remained in place and did not encroach on the AOE-induced changes in compartment size (AOE2 condition illustrated in Figure 3A). In this condition, the nodal compartment had the same altered dimensions as the nodal compartment in AOE1. However, the expression of I_Na_ and I_HT_ remained the same as the control condition. In AOE2 the regions n1 contained no voltage dependent channels and were sized such that the combined values of the two n1 regions and the nodal region equaled the value of the nodal region in Table 1. Similar calculations were carried out for the PN and JP and considered the PN length decrease the JP length increase after AOE (Tagoe et al., 2014). Each INR was divided into compartments such that the length of each compartment was less than 0.1 λ (length constant), to ensure each compartment was isopotential, the generally accepted practice in such simulations (Carnevale and Hones, 2006; Sterratt et al., 2011). The length constant (λ) was calculated as:

$$\lambda =\sqrt{\frac{\text{rad}*{\text{R}}_{\text{m}}}{\text{2}*{\text{R}}_{\text{a}}}}$$

where rad is the axon radius, R_a_ is the axoplasmic resistance and R_m_ is the membrane resistance.

### Passive membrane properties

The basic passive properties and the methods used to calculate these properties have previously been described in detail (Kolaric et al., 2013). The value of axoplasmic resistance (Ra) was 70 Ω.cm throughout. Cm was assumed to be 1 μF.cm^−2^ and nodal g_L_ was 0.2 mS.cm^−2^. In the model the control axon had 23.7 lamella wraps of myelin, whereas AOE decreased the number to 15.8 (Tagoe et al., 2014). The values were rounded-up to the nearest whole number to ease computations.

### Voltage-dependent conductances

The model described in Hodgkin and Huxley (1952a,b,c) contained a fast sodium current (I_Na_), a high threshold (I_HT_) Kv3.1 potassium current, a low threshold (I_LT_) Kv1.1 potassium current and a leak current (I_L_). I_Na_ and I_HT_ were located at the nodal regions and I_LT_ was located at the JPN (7).

The voltage dependence of the I_HT_ was described by:

$${\text{I}}_{\text{HT}}={\text{g}}_{\text{K}}{\text{n}}^{3}(1-\gamma +\gamma \text{p})({\text{V}}_{\text{m}}-{\text{V}}_{\text{HT}})$$

where 0 > γ > 1, and the variables *p* and *n* are described by dj/dt = α_j_(1 − j) − β_j_ where *j* = *n* or *p*, and is calculated by α_j_ = k_αj_ exp(η_αj_.V) and β_j_ = k_βj_ exp(η_βj_.V).

k_αj_, k_βn_, η_αj_ and η_βj_ determine the rate and voltage dependence respectively of current activation with the values required to calculate the rate constants contained in Table 3.

**Table 3:** **Rate constant parameters of potassium and sodium conductances**.

	**α_n_**	**β_n_**	**α_p_**	**β_p_**
k_(IHT)_	0.2719 ms^−1^	0.1974 ms^−1^	0.00713 ms^−1^	0.0935 ms^−1^
η_(IHT)_	0.04 mV^−1^	0 mV^−1^	–0.1942 mV^−1^	0.0058 mV^−1^
	**α_l_**	**β_l_**	**α_r_**	**β_r_**
k_(ILT)_	1.2 ms^−1^	0.2248 ms^−1^	0.0438 ms^−1^	0.0562 ms^−1^
η_(ILT)_	0.03512 mV^−1^	−0.0319 mV^−1^	−0.0053 mV^−1^	−0.0047 mV^−1^
	**α_m_**	**β_m_**	**α_h_**	**β_h_**
k_(INa)_	76.4 ms^−1^	0.0381 ms^−1^	0.00013 ms^−1^	1.999 ms^−1^
η_(INa)_	0.037 mV^−1^	−0.043 mV^−1^	−0.1216 mV^−1^	0.0384 mV^−1^

Rate constant parameters of the voltage dependent conductances derived from historical data Kanemasa et al. (1995). (IHT) and (ILT) refer to the high and low threshold K^+^ currents respectively. The rate constants are calculated based on the terminology used by Kanemasa et al. (1995), where α_n_ = k_αj_ exp(η_αj_.V) and β_n_ = k_βj_ exp(η_βj_ V) etc.

The voltage dependent currents I_Na_ and I_LT_ were described by:

$$\begin{array}{l} {\text{I}}_{\text{Na}}={\text{g}}_{\text{Na}}{\text{m}}^{3}\text{h}({\text{V}}_{\text{m}}-{\text{V}}_{\text{Na}})\\ {\text{I}}_{\text{LT}}={\text{g}}_{\text{K}}\text{lr}({\text{V}}_{\text{m}}-{\text{V}}_{\text{LT}}) \end{array}$$

where I is the current per unit area and g is the voltage dependent conductance where g_Na_ = 0.05 S.cm^−2^, g_HT_ = 0.015 S.cm^−2^ and g_LT_ = 0.002 S.cm^−2^. V_m_ is the membrane potential and V refers to the reversal potential whereby V_HT_ = V_LT_ = −80 mV and V_Na_= 50 mV.

The leak current was calculated as I_L_ = g_L_(V_m_ – V_L_), where g_L_ = 0.2 mS.cm^−2^ and V_L_ = −63 mV.

The variables *m, h, r* and *l* are associated with channel activation (*m, l*) and inactivation (*h, r*) and are calculated as dj/dt = α_j_(1 − j) − β_j_ where *j* = l, *r, m* or *h*. The rate and voltage dependence respectively of I_Na_ and I_LT_ are calculated in a similar manner to I_HT_ with the parameters for calculating I_LT_ and I_Na_ contained in Table 3.

### Conduction velocity

The model comprised 7 alternating nodal and internodal regions and conduction velocity was calculated between the first (node 0) and last node (node 6). When the conduction velocity was reported in function of the number of INRs affected (Figure 3D), the conduction velocity at node 0 was equivalent to the control condition.

## Results

### Action potential conduction

The model comprising 7 alternating nodal and internodal regions was capable of firing action potentials in response to injected current. Action potentials were recorded at 6 sequential nodes and data clearly showed that action potentials were propagating from node to node (Figure 2B). As we have no measure of INR length (INL) from our morphological data we estimated 100 μm as a reasonable guess as this lies between values from two previous reports (Rattay et al., 2001; Smit et al., 2010). The conduction velocity in control condition was calculated as 5.45 ms^−1^. (with an INL of 100 μm and a calculated length constant (λ) for the control axon of about 200 μm, Figure 2C). Increasing INL caused a decrease in conduction velocity as the INL extended beyond 100 μm (Figure 2C).

### AOE-induced decrease in conduction velocity

We adjusted the model to take into account the morphological changes that occur because of AOE. In our first simulation of the effects of AOE (AOE1) we altered the current density that would result from altered distribution of channels along the node and JPN (see Methods for details). We found that there was a significant effect on action potential propagation (Figure 3B) compared to control (Figure 2B) resulting in a decreased conduction velocity (Figures 3D,E). The results illustrated in Figure 3D derive from simulations where we imposed the AOE-induced dysmyelination on increasing numbers of adjacent INRs in the model. Incrementally increasing the number of INRs affected by AOE dysmyelination showed a non-linear decrease in conduction velocity that steeply decreased with increasing number of INRs affected, as previously described in the corpus callosum model (Kolaric et al., 2013).

In the second simulation we modeled unchanged current density and channel distribution as a result of AOE (AOE2, see Methods). Similarly to AOE1, we found that there was a significant effect on action potential propagation (Figures 3B,C) compared to control condition (Figure 2B). Conduction velocity was similarly decreased in AOE2 and AOE1 (Figure 3E) and the decreased conduction velocity was more apparent as the number of adjacent INRs affected increased (Figure 3D).

### Absence of effect of AOE on firing frequency

**Figure 4:**
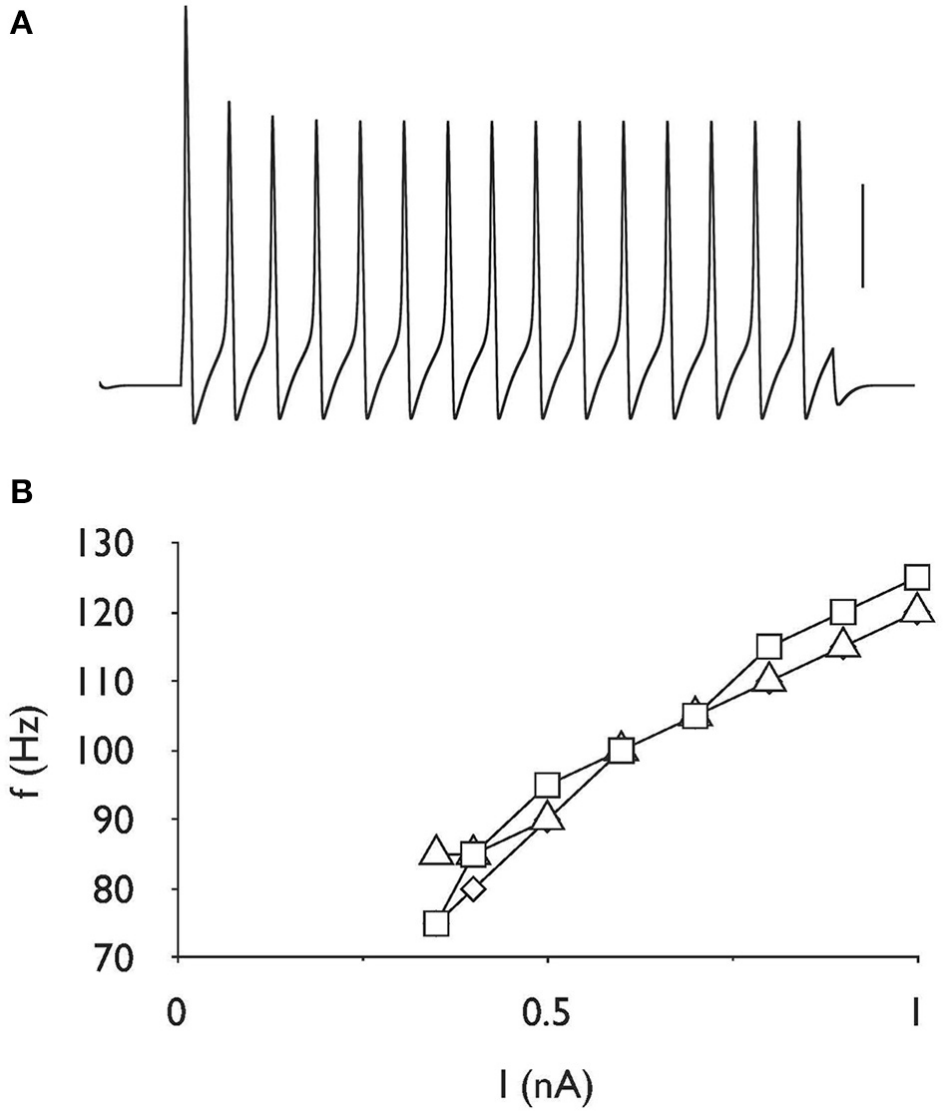
**Firing rate is unchanged after AOE**. **(A)** Action potentials evoked by a current of 0.35 nA for 200 ms, demonstrating the capacity for repetitive firing in the control model. Scale bar is 50 mV and the duration of the entire trace is 250 ms. **(B)** Stimulus intensity versus frequency response showing linear increase in firing frequency in response to increasing stimulus current up to 1 nA. There is little difference in the firing frequency properties for control (□), AOE 1 (◊) or AOE 2 (▵) treatment. Note y-axis starts at 70 Hz.

Repetitive firing was induced in the control model by injecting a current pulse of 0.35 nA for a duration of 200 ms (Figure 4A). The resulting action potentials showed regular firing pattern, i.e., a lack of adaptation. Increasing the stimulus intensity resulted in increased frequency of firing, although at higher stimulus intensities the action potentials became much smaller. Imposing the same stimulus on the AOE1 and AOE2 models had a negligible effect of firing frequency (Figure 4B).

### Effects of GNA expression on conduction velocity

**Figure 5:**
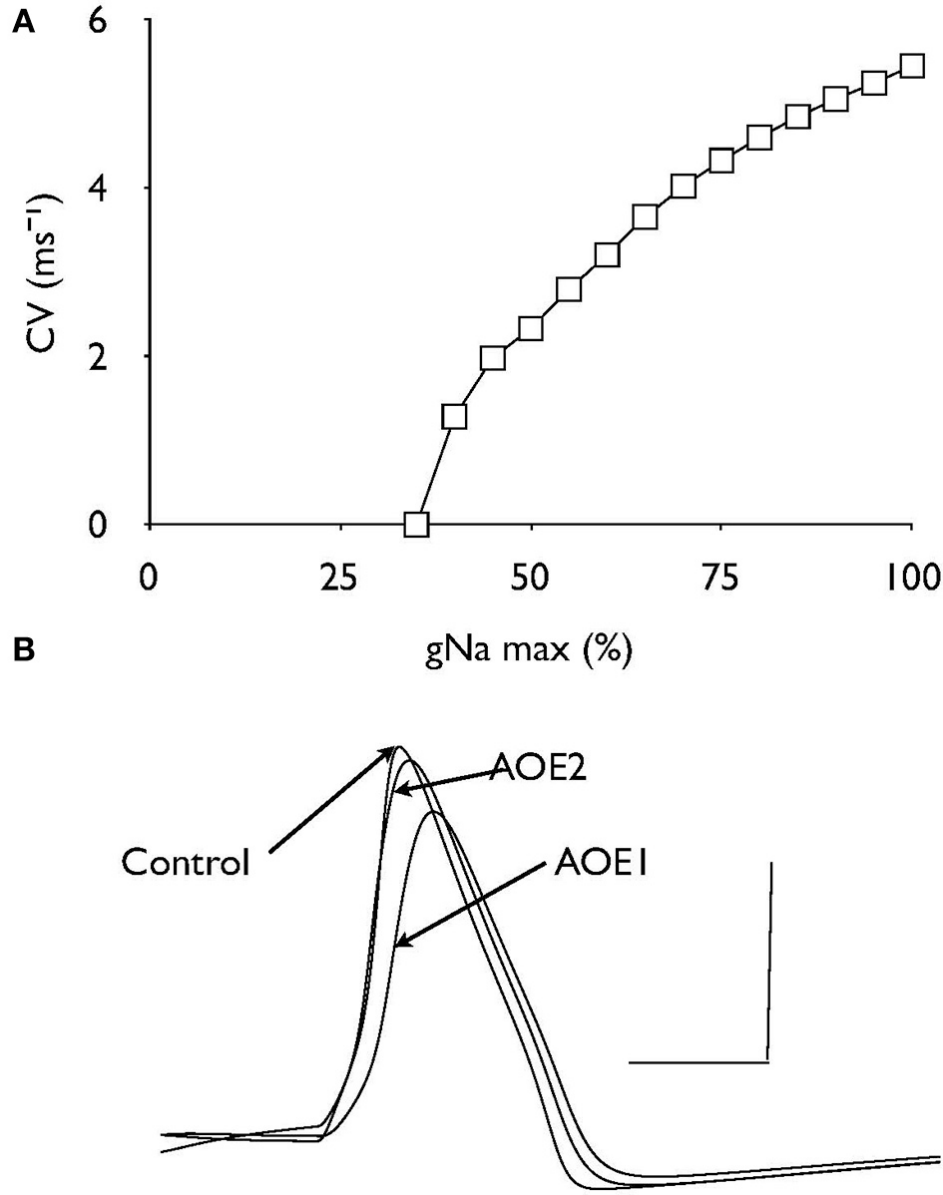
**The effect of gNa on conduction velocity**. **(A)** Decreasing the value of gNa relative to the control value as 100% resulted in a non-linear decrease in conduction velocity. **(B)** Nodal recordings of gL under control and AOE treated conditions. Scale bars 0.01 nA and 1 ms.

Decreasing the density of Na^+^ channel expression by attenuating the value of gNa caused a non-linear decrease in conduction velocity until conduction block occurred at a value of about 35% of control (Figure 5A). The passive leak conductance across the node (gL) under control and both AOE conditions are superimposed to show there are slight effects on gL as a result of AOE but these differences are minimal (Figure 5B) and do not indicate increased shunting of current via the node.

## Discussion

The present computer simulations are based on our previous morphological data on the AN (Tagoe et al., 2014). We have shown that the conduction velocity was dependent on the INR length as previously described in the corpus callosum model (Kolaric et al., 2013) and this over a range of between 0.05 and 0.3 mm. Our estimated conduction velocities of the AN in the range of 2–12 m.s^−1^ were similar to values (6–14 m.s^−1^) reported for the AN (Poma et al., 2008; Imennov and Rubinstein, 2009). Huxley and Stampfli (1949) suggested that the conduction velocity in myelinated nerve fibers should reach a maximum at a particular internode distance, and that the maximum should be relatively flat (Huxley and Stampfli, 1949). We also found that the conduction velocity in myelinated AN fibers did not increase linearly with increasing internodal length and reached a plateau at an internodal length of 0.3 mm. Our previous field potential recordings of the AN estimated its conduction velocity at 3 m.s^−1^ (Tagoe et al., 2014). Although this lower range of values could be due to an AN internodal length of around 200 μm, it is likely to be due to the recruitment of lower conduction velocity fibers while recording compound action potentials.

Our previous study showed that dysmyelination of the AN resulted in the decrease of the myelin thickness, a marked elongation of nodes of Ranvier and juxtaparanodes, and a retraction of paranodes, slowing the AN conduction velocity by about 3-fold (Tagoe et al., 2014). Using the same morphological values as in Tagoe et al. (2014) and keeping the internodal length of 100 μm (Rattay et al., 2001; Smit et al., 2010) constant, we have reproduced here a 3-fold slowing of conduction velocity. This result was anticipated considering that the elongation of the node would increase its capacitance and decrease its resistance, and therefore increase the time to reach the threshold contributing to lower conduction velocity (Hartline and Colman, 2007). The decreased conduction velocity was more apparent with the number of INR affected, as the increased nodal surface area resulted in a decreased current density, increased nodal and INR capacitances (Table 2). As the conduction velocity decreases with increasing length of dysmyelinated axon, we would anticipate conduction block for distances exceeding 700 μm (Figure 3D) as a significant length of axon would be affected. Conduction block has indeed been observed for distances exceeding 1 mm (Tagoe et al., 2014).

Nodal clusters of voltage-gated Na^+^ channels are lost after demyelination, but reappear after remyelination (Dugandzija-Novakovic et al., 1995; Novakovic et al., 1996). Similarly, juxtaparanodal Kv1.1 and Kv1.2 channels disperse after demyelination and reorganize with remyelination (Rasband et al., 1998). Deficits of myelin subdomains along the AN could therefore alter spatial segregation of voltage-gated channels and impair the action potential propagation (Peles and Salzer, 2000; Hossain et al., 2005). However we found that spatial segregation of nodal Na^+^ and Kv3.1 channels or juxtaparanodal Kv1.1 channels had a minimal impact on AN conduction velocity. Axonal targeting of Kv3.1 channels is critical to enable neurons to fire action potentials at the maximal frequency (Gu et al., 2012). Our study further suggests that the re-localization of Kv3.1 channels is unlikely to affect action potential firing frequency during AN dys-myelination. Axonal demyelination has been shown to decrease nodal expression of I_Na_ (Craner et al., 2004). Our computational analysis indicates that decreasing the Na^+^ channel density has a negative impact on the conduction velocity and that decreasing the Na^+^ channel density by 65% relative to control values can fully account for the conduction block observed after AOE.

In conclusion the AOE induced morphological alterations of myelin sub-domains and changes of density of Na^+^ channels are the likely cause of the decreased conduction velocity of the AN and the conduction block observed for distances exceeding 1 mm (Tagoe et al., 2014)

### Conflict of interest statement

The authors declare that the research was conducted in the absence of any commercial or financial relationships that could be construed as a potential conflict of interest.
